# Supplementary material for: Management of Common Infections in German Primary Care: A Cross-Sectional Survey of Knowledge and Confidence among General Practitioners and Outpatient Pediatricians
Source: Antibiotics (Basel). 2021 Sep 20;10(9):1131. doi: 10.3390/antibiotics10091131 (PMC8466449; doi:10.3390/antibiotics10091131)
Supplement: Supplementary file 1 [file antibiotics-10-01131-s001.zip › Survey 1.pdf]

Please mark like this: ☐ ☒ ☐ ☐ ☐

Corrections: ☐ ■ ☐ ☒ ☐

1. Information on your own handling of antibiotics

There is currently a lot of talk in experts and in the public about the frequent prescription of antibiotics. We would like to know about your experience with antibiotics in everyday life.

1.1	I feel confident when dealing with antibiotics in general.	I don't agree at all.	☐	☐	☐	☐	☐	☐	☐	I totally agree.
1.2	I feel confident to decide whether antibiotics are indicated for the treatment of common infectious diseases in the practice or not.	I don't agree at all.	☐	☐	☐	☐	☐	☐	☐	I totally agree.
1.3	I feel confident in the choice of an antibiotic agent for the treatment of common infectious diseases.	I don't agree at all.	☐	☐	☐	☐	☐	☐	☐	I totally agree.
1.4	I feel confident when determining the duration of therapy for an antibiotic for common infectious diseases.	I don't agree at all.	☐	☐	☐	☐	☐	☐	☐	I totally agree.
1.5	I feel confident to dose an antibiotic when treating common infectious diseases.	I don't agree at all.	☐	☐	☐	☐	☐	☐	☐	I totally agree.

2. How would you rate the following statements: are they right or wrong?

2.1	Purulent, yellow sputum when coughing indicates the bacterial genesis of an infection and is an indication for antibiotic treatment.	☐ Right	☐ Wrong
2.2	Cefuroxim (2nd generation cephalosporine) more often leads to the development of antibiotic resistances than Cefaclor (1st generation cephalosporine).	☐ Right	☐ Wrong
2.3	An erythema chronicum migrans should be treated with Doxycycline 200mg / 1 per day.	☐ Right	☐ Wrong
2.4	The likelihood of resistance development increases with the duration of antibiotic treatment.	☐ Right	☐ Wrong
2.5	In the treatment of infections, a longer therapy than recommended in the guidelines leads to better treatment results.	☐ Right	☐ Wrong
2.6	When treating wound infections, Clindamycin is preferable to other antibiotics because of its good tissue penetrance.	☐ Right	☐ Wrong
2.7	Aminopenicillins increase the likelihood of Clostridium difficile-associated diarrhea to the same extent as cephalosporins.	☐ Right	☐ Wrong
2.8	If Trimethoprim is used for the three-day therapy of uncomplicated cystitis, 2x200mg/day should be administered.	☐ Right	☐ Wrong
2.9	If an acute uncomplicated cystitis is to be treated with antibiotics, quinolones are **not** among the therapeutic agents of first choice.	☐ Right	☐ Wrong
2.10	For the antibiotic treatment of bacterial tonsillitis, a broad spectrum penicillin (e.g. AmoxiClav) should be selected in order to treat other potential pathogens in addition to *Streptococcus pyogenes.*	☐ Right	☐ Wrong

2. How would you rate the following statements: are they right or wrong? [Fortsetzung]

2.11 When treating a community-acquired pneumonia without complications in an otherwise healthy adult, cephalosporins should be preferred to aminopenicillins for antibiotic therapy.	☐ Right	☐ Wrong
2.12 The dose of antibiotics for children must be converted into ml of the corresponding liquid formula to be given based on their body weight.	☐ Right	☐ Wrong
2.13 The dosage of Doxycycline must be adjusted to the glomerular filtration rate (GFR) in the presence of renal insufficiency.	☐ Right	☐ Wrong
2.14 Amoxicillin is suitable for the treatment of an erythema chronicum migrans even in pregnant women and children under the age of 9.	☐ Right	☐ Wrong
2.15 Otitis media in children under the age of 6 should primarily be treated with antibiotics to avoid complications.	☐ Right	☐ Wrong
2.16 In the treatment of upper respiratory tract infections antibiotics should not usually be prescribed, as they are mostly viral infections.	☐ Right	☐ Wrong
2.17 Female patients with asymptomatic bacteriuria should be treated with antibiotics to prevent the transition to a symptomatic urinary tract infection.	☐ Right	☐ Wrong
2.18 If a tonsillitis is treated with oral Penicillin V, Penicillin V should be administered for a period of 7 days.	☐ Right	☐ Wrong
2.19 If antibiotic therapy is indicated for sinusitis, Amoxicillin 2x500mg/day over the recommended treatment period is suitable.	☐ Right	☐ Wrong
2.20 In the case of mild to moderate pneumonia in a patient without comorbidities, a therapy duration of 5-7 days is usually sufficient and can be shortened when the condition of the patient improves rapidly.	☐ Right	☐ Wrong

3. When treating infectious diseases, I follow...

3.1	... the guideline recommendations of my specialist society.	I don't agree at all.	☐	☐	☐	☐	☐	☐	☐	I totally agree.
3.2	... my clinical experience.	I don't agree at all.	☐	☐	☐	☐	☐	☐	☐	I totally agree.
3.3	... the recommendations from colleagues.	I don't agree at all.	☐	☐	☐	☐	☐	☐	☐	I totally agree.
3.4	... the specialist information provided by the drug manufacturer.	I don't agree at all.	☐	☐	☐	☐	☐	☐	☐	I totally agree.
3.5	... the patients' wishes.	I don't agree at all.	☐	☐	☐	☐	☐	☐	☐	I totally agree.

4. To what extent do the following situations influence your prescription behaviour for antibiotics?

4.1	When I find the patient to be very demanding during the consultation, I sometimes give in and prescribe an antibiotic without it being necessarily indicated.	not at all	☐	☐	☐	☐	☐	☐	☐	very strongly
4.2	When I am concerned that the patient's symptoms might worsen, for example over the weekend, I sometimes prescribe an antibiotic without it being necessarily indicated.	not at all	☐	☐	☐	☐	☐	☐	☐	very strongly
4.3	When the waiting room is full, I sometimes prescribe an antibiotic without it being necessarily indicated, in order to make the patient feel good despite the short consultation time.	not at all	☐	☐	☐	☐	☐	☐	☐	very strongly

5. Information on training courses

5.1 Have you participated in one or more advanced training courses on *(rational) antibiotic therapy* within the last 3 years?
☐ Yes ☐ No

5.2 Would you like more training on *(rational) antibiotic therapy*?
☐ Yes ☐ No

5.3 How sufficient do you perceive the offer for advanced training courses on the subject of *(rational) antibiotic therapy*?
not at all ☐ ☐ ☐ ☐ ☐ ☐ ☐ fully

5.4 What kind of training would you prefer and where? (**Please select 2 options.**)

☐ online course
☐ single short courses (about 1.5 hours)
☐ longer block course on the weekend (about 12 hours)
☐ near the place of residence
☐ distance to home up to 100km
☐ distance to home more than 100km

5.5 If your answer was not mentioned under 5.4, you have the opportunity to contribute your own ideas here:

6. Personal information

6.1 Your gender: ☐ male ☐ female ☐ other

6.2 Please indicate your age.
☐ 20 to 29 years ☐ 30 to 39 years ☐ 40 to 49 years
☐ 50 to 59 years ☐ 60 to 69 years ☐ 70 years or older

6.3 Location of the practice (count of inhabitants)
☐ rural (<5.000) ☐ small town (5.000-20.000) ☐ city (20.001-100.000)
☐ big city (>100.000)

6.4 How long have you been practicing as a doctor?
☐ less than 5 years ☐ 5 to 14 years ☐ 15 to 24 years
☐ 25 to 34 years ☐ 35 to 44 years ☐ 45 to 54 years
☐ more than 55 years

6.5 How long have you been working as a general practitioner?
☐ less than 5 years ☐ 5 to 14 years ☐ 15 to 24 years
☐ 25 to 34 years ☐ 35 to 44 years ☐ 45 to 54 years
☐ more than 55 years

6.6 What is your position in your practice?
☐ employed doctor ☐ doctor in further training in general practice ☐ self-employed practice owner in joint practice
☐ equal partner in joint practice

6.7 What is the structure of the practice you're working in?
☐ individual practice ☐ joint practice ☐ medical care center

6.8 What is the number of doctors in the practice you're working in? **(including yourself)**
