# Supplementary figures and images for: Management of Common Infections in German Primary Care: A Cross-Sectional Survey of Knowledge and Confidence among General Practitioners and Outpatient Pediatricians
Source: Antibiotics (Basel). 2021 Sep 20;10(9):1131. doi: 10.3390/antibiotics10091131 (PMC8466449; doi:10.3390/antibiotics10091131)

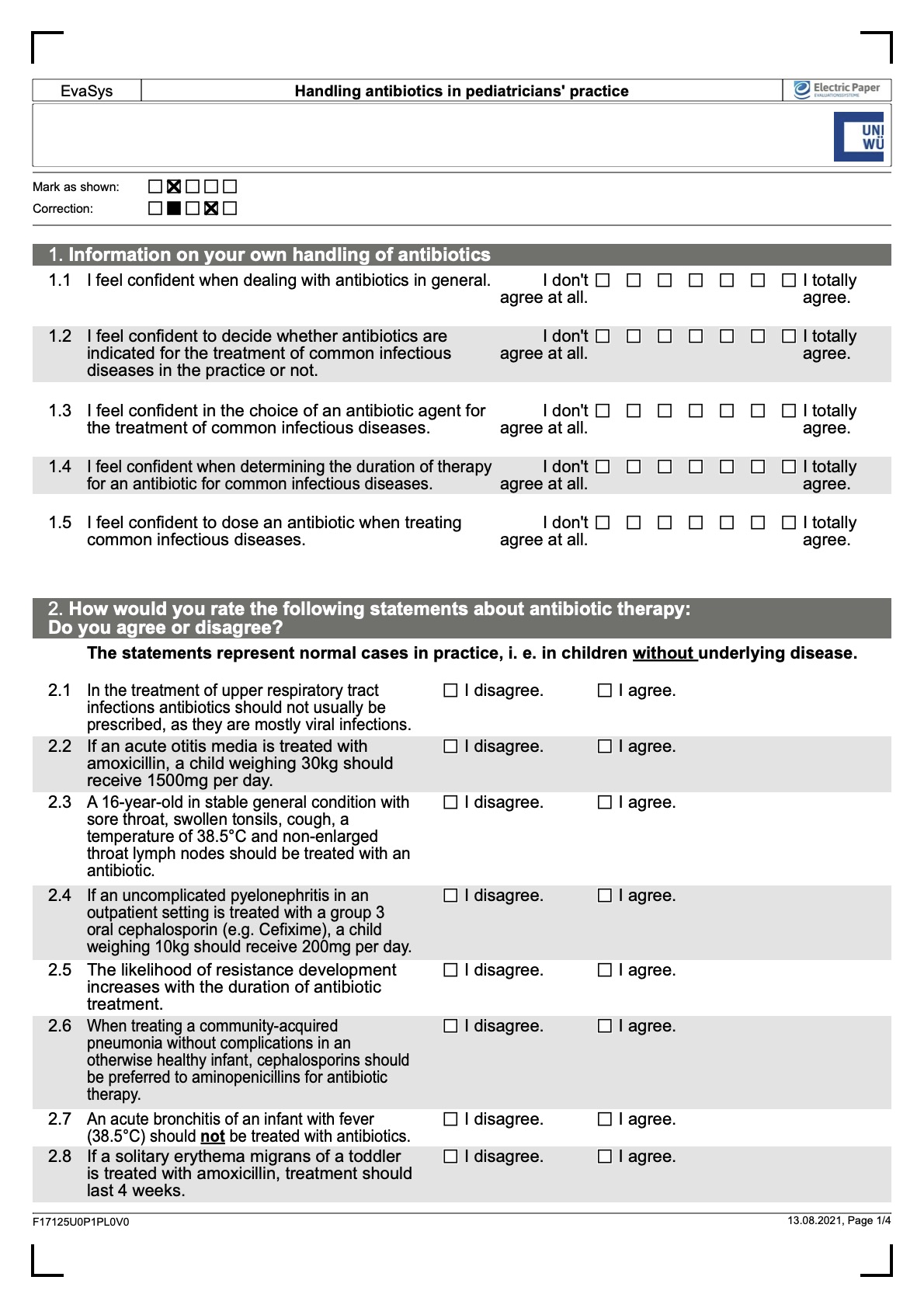

Supplement: Supplementary file 1 [file antibiotics-10-01131-s001.zip › Survey 2.jpg]
